# Developmental Impacts of Epigenetics and Metabolism in COVID-19

**DOI:** 10.3390/jdb12010009

**Published:** 2024-02-09

**Authors:** Noopur Naik, Mansi Patel, Rwik Sen

**Affiliations:** 1 Department of Molecular, Cellular & Developmental Biology, University of Colorado Boulder, Boulder, CO 80309, USA; nxn175@case.edu; 2 Institute of Genomics and Integrative Biology, Delhi 110007, India; mansipatel241002@gmail.com; 3 Active Motif, Inc., Carlsbad, CA 92008, USA

**Keywords:** epigenetics, metabolic reprogramming, systemic toxicity, diabetes mellitus, hyperglycemia, glucose metabolism, inflammation, COVID-19, immunometabolic, vitamin D

## Abstract

Developmental biology is intricately regulated by epigenetics and metabolism but the mechanisms are not completely understood. The situation becomes even more complicated during diseases where all three phenomena are dysregulated. A salient example is COVID-19, where the death toll exceeded 6.96 million in 4 years, while the virus continues to mutate into different variants and infect people. Early evidence during the pandemic showed that the host’s immune and inflammatory responses to COVID-19 (like the cytokine storm) impacted the host’s metabolism, causing damage to the host’s organs and overall physiology. The involvement of angiotensin-converting enzyme 2 (*ACE2*), the pivotal host receptor for the SARS-CoV-2 virus, was identified and linked to epigenetic abnormalities along with other contributing factors. Recently, studies have revealed stronger connections between epigenetics and metabolism in COVID-19 that impact development and accelerate aging. Patients manifest systemic toxicity, immune dysfunction and multi-organ failure. Single-cell multiomics and other state-of-the-art high-throughput studies are only just beginning to demonstrate the extent of dysregulation and damage. As epigenetics and metabolism directly impact development, there is a crucial need for research implementing cutting-edge technology, next-generation sequencing, bioinformatics analysis, the identification of biomarkers and clinical trials to help with prevention and therapeutic interventions against similar threats in the future.

## 1. Introduction

Epidemics and the COVID-19 pandemic are major threats to the global population. Viral infections spread rapidly among populations, with often lethal outcomes. The “black death” bubonic plague of the 14th century, the smallpox pandemic, the yellow fever infection of the 16th century, the dengue viral fever of the 18th century, the epidemic of Human Immunodeficiency Virus (HIV) and, most recently, the COVID-19 pandemic caused by Severe Acute Respiratory Syndrome Coronavirus 2 (SARS-CoV-2) are all examples of such disastrous outbreaks on our planet [1]. COVID-19 emerged towards the end of 2019, when it appeared in the form of an epidemic, but, by early 2020, it had spread worldwide. Reports showed increased frequencies of SARS-CoV-2 infection-related morbidities, which were so extreme that, in March 2020, the World Health Organization (WHO) declared COVID-19 as a pandemic, causing a global crisis [2]. The current death toll from COVID-19 is above 6.96 million. Furthermore, epigenetic and metabolic abnormalities associated with viral infections have shown devastating consequences for COVID-19 cases.

According to the CDC, or the Centers for Disease Control and Prevention, in the United States, there are several symptoms of COVID-19, which can be accessed at https://www.cdc.gov/coronavirus/2019-ncov/symptoms-testing/symptoms.html (accessed on 8 February 2024). A cough, headache, shortness of breath, fever or chills, muscular or body pains, altered or lost taste and smell, sore throat, congestion, nausea or vomiting and runny nose are some of the most frequent symptoms listed for COVID-19. Another risk is posed by the SARS-CoV-2-related secondary infections of *Rhizopus* or *Aspergillus*-related mucormycosis. COVID-19-associated mucormycosis (CAM) is known to affect those that have a weakened immune system, probably due to underlying health comorbidities like diabetes, organ transplantation or chemotherapy [3]. Studies show that COVID-19 patients have epigenetic alterations leading to aberrant gene expression. Early clinical data on COVID-19 showed that individuals with type 2 diabetes mellitus (T2DM) and other metabolic disorders that impair general metabolic health were more likely to experience a more severe infection course than individuals who were metabolically fit prior to contracting the virus [4].

Immune responses are triggered during infections, which can affect various epigenetic and metabolic processes. This is because infections cause epigenetic manipulations through altered gene expression and protein production, in addition to prompting metabolic changes that increase cellular energy to enhance the immune response. DNA methylation is one of the major epigenetic modifications that regulates gene expression. A recent study on the hearts and kidneys of COVID-19 patients 7 days after infection showed the altered methylation of DNA at 172 sites in the kidneys and 49 sites in the heart [5]. The abnormal epigenetic signature is a major factor contributing to the aberrant gene expression leading to multi-organ failure in COVID-19. Another study on T-cells unique to acute COVID-19 patients revealed the upregulation of metabolic protein VDAC1 (voltage dependent anion channel 1) and H3K27me3, which is a major histone modification marking transcriptional repression [6]. The epigenetic and metabolic implications of COVID-19 significantly affect aging, as indicated by a genome-wide DNA methylation analysis of severe COVID-19 patients, who showed accelerated biological aging compared to non-severe COVID-19 patients. Furthermore, even non-severe COVID-19 patients show significantly higher levels of aging compared to healthy individuals. These studies underline the importance of identifying the epigenetic and metabolic markers associated with viral diseases, as they provide potential therapeutic targets. In this direction, further research on epigenetics, metabolomics and other omics methodologies is needed, along with the ability to process high-throughput samples from large cohorts.

High-throughput omics methodologies require advanced, cutting-edge tools to be successful and precise. Epigenetic methods like DNA methylation, ChIP-seq, ATAC-seq, transcriptomics technique like RNA-seq, and proteomics workflows are used for the high-throughput analysis of samples from COVID-19 patients. For proteomics, a review by Santorelli et al. highlights the advantages of cross-linking mass spectrometry (XL-MS), which is superior to conventional methods to analyze the dynamic interactomics of the cellular landscape [7]. Development of technology like pixelated ultrasound for consistent and high-throughput sample preparation, which integrate conveniently with epigenetics, genomics, transcriptomics and proteomics workflows, is also likely to contribute towards the success of research in the above areas that provide well-rounded multi-level omics information [8]. Collectively, these methods will improve our understanding of COVID-19 at the level of multiomics. For metabolomics, a recent study using targeted metabolomics with tandem mass spectrometry, on serum from 52 COVID-19 patients categorized by severity, revealed the levels of metabolites in the samples [9]. The study showed how bioinformatics and correlational analysis could help to identify dysregulated pathways and connections between metabolite profiles and inflammation leading to organ damage.

Multiomics studies provide multifarious information towards understanding a disease. Despite the recent discoveries, the field is not well explored. This review aims to elaborate on the impact of COVID-19 on epigenetics, inflammation and metabolism, including insulin resistance, diabetes, impaired lipid metabolism and obesity. This review also aims to address the relationship between epigenetics and COVID-19, in the context of both metabolic reprogramming and potential therapeutic targets. Collectively, the information will benefit the understanding of how the epigenetic, metabolic and developmental complications of COVID-19 are inter-related, because epigenetics and metabolism directly impact development.

## 2. SARS-CoV-2 Induces Metabolic Reprogramming and Epigenetic Changes

Although SARS-CoV-2 is a respiratory pathogen, its extrapulmonary involvement causes significant issues in other organ systems, such as metabolic complications and immune dysfunction, in addition to increased morbidity and mortality [10,11]. Hence, understanding the pathogenesis of extrapulmonary involvement and the resulting systemic toxicity could fill critical gaps in our understanding of the disease presentation of COVID-19. A number of factors are hypothesized to contribute to systemic toxicity in COVID-19. For example, ACE2 (the functional receptor for SARS-CoV) is expressed in multiple extrapulmonary tissues [12] that are damaged as the result of viral infection, the subsequent inflammatory immune response with systemic cytokine release or the “cytokine storm”, which are possible mechanisms of injury [10].

To distinguish the pathogenesis of systemic complications of COVID-19, a study was performed where a murine model expressing the human *ACE2* transgene in multiple tissues was generated, and *n* = 5 transgenic mice were administered SARS-CoV-2 [13]. As controls, a group of *n* = 5 transgenic mice was not administered SARS-CoV-2, and a group of *n* = 5 mice without the human *ACE2* transgene underwent SARS-CoV-2 administration. At 7 days after systemic SARS-CoV-2 infection, mice in the experimental group developed a distinct phenotype that aligned with human COVID-19 presentation. This included severe weight loss, morbidity, neutrophilia, lymphopenia, splenic atrophy, myofibrillar disarray and myocardial edema.

To better understand organ-related complications in COVID-19, organs were harvested for bulk RNA sequencing 3 days after infection (1 day prior to systemic toxicity onset) and at 7 days after infection. At 3 days post-infection, pathways related to interferon (IFN) and cytokine-mediated signaling were enriched, indicating an antiviral immune response. The response was no longer evident at 7 days post-infection, although the expression of genes regulating oxidative phosphorylation and the electron transport chain (ETC) was decreased in multiple organs. These results suggest that disease pathogenesis and the development of morbidity are associated with temporal transcription patterns [13].

Due to the close connections between the tricarboxylic acid cycle (TCA) and the ETC, TCA gene regulation across four organs was examined in a study. The downregulation of the TCA cycle genes was consistent across the heart, lung, kidney and spleen. In line with these observations, metabolomic profiling confirmed the lower TCA cycle metabolite levels in the serum of the experimental group. Finally, DNA methylation analysis of the heart and kidney at 7 days post-infection was performed to investigate whether epigenetic changes could contribute to gene expression in multiple organs. This revealed differentially methylated sites in the heart (172 sites) and kidney (49 sites), suggesting that tissue-specific epigenetic changes occur soon after SARS-CoV-2 infection [13]. Other studies have also reported the adverse effects of COVID-19 on the heart [14]. These findings help us to better understand COVID-19’s disease presentation, suggesting that SARS-CoV-2 induces metabolic reprogramming and epigenetic changes, which may contribute to systemic toxicity.

## 3. COVID-19 Is Associated with Accelerated Epigenetic Aging and Hence Development

Chronological age is an established and independent [15] risk factor of severity and death in COVID-19 patients [16,17]. Additionally, epigenetic studies have indicated that markers of biological age, like DNA methylation (DNAm), are associated with severe COVID-19 [18]. The disparity between biological and chronological age (epigenetic age acceleration) has been related to survival outcomes in age-related diseases [19]. However, little is known about epigenetic aging during severe vs. non-severe COVID-19, and whether it could predict disease severity and model disease progression.

To assess the association between accelerated epigenetic aging and SARS-CoV-2 infection severity, a genome-wide DNA methylation study was conducted on the whole blood samples of 232 healthy individuals, 194 individuals with non-severe COVID-19 and 213 individuals with severe COVID-19 [5]. The epigenetic age acceleration of individuals was calculated by determining the difference between their chronological age and epigenetic age (calculated by applying epigenetic clocks and telomere length estimators (DNAmTL) to an individual’s methylation profile). Individuals with severe COVID-19 showed significant DNAm age acceleration and DNAmTL attrition acceleration compared to individuals with non-severe COVID-19. In comparison, individuals with non-severe COVID-19 had significantly higher DNAm and DNAmTL acceleration compared to healthy individuals [5].

To further understand these associations, a study analyzed the dynamic acceleration of epigenetic aging in six individuals with COVID-19 and six uninfected controls across different disease phases, defined by inflammatory markers and temporal disease severity [20]. Increasing age acceleration was observed in the initial disease phases, which could then be partially reversed in the later phases, although the mechanism behind this observation requires further research. Overall, this study suggests that COVID-19 may accelerate epigenetic aging and that markers of accelerated epigenetic aging could help to predict disease progression [21] and identify patients with a higher risk of developing severe COVID-19. Accelerated epigenetic aging indicates dysregulation in development, which is yet another connection between COVID-19-mediated abnormalities of developmental biology and epigenetics.

## 4. Epigenetic Regulation of Viral Pathogenicity Suggests Epi-Drugs as a Therapeutic Approach against COVID-19

During infection, SARS-CoV-2 uses transmembrane serine protease 2 (*TMPRSS2*) and the *ACE2* receptor to infect the host cell [22,23] and the RNA polymerase to synthesize viral proteins [24]. As a response, the host immune system causes a “cytokine storm”, which can lead to an uncontrolled inflammatory response [25], culminating in lung injury, acute respiratory distress syndrome and organ failure. Epigenetic alterations such as DNA methylation have crucial roles in most biological processes and modify genetic expression to allow cells to adapt to environmental changes [26]. For example, epigenetic pathways could impact the expression of genes like *ACE2* and immunoregulatory genes involved throughout COVID-19’s pathogenesis [26,27]. Epigenetic pathways may be altered by SARS-CoV-2 and are also linked to COVID-19 severity [28]. Hence, further insights into epigenetics would allow the possibility of more precise therapies against COVID-19.

**Figure 1:**
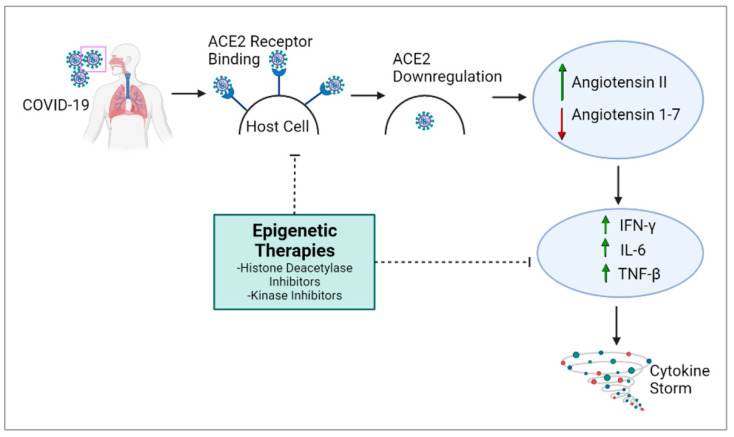
Epigenetic therapies can inhibit biological processes that play a role in generating the cytokine storm in COVID-19. The therapies can target *ACE2*, *TMPRSS2* and Furin. IFN—Interferon, IL—Interleukin, TNF—Tumor Necrosis Factor, ↑—upregulation, ↓—downregulation.

The overexpression of *ACE2* is associated with higher COVID-19 severity [29], and epigenetic processes are responsible for *ACE2* control and expression [26]. Histone deacetylase (HDAC) can also the modulate epigenetic effects on COVID-19: HDAC upregulates *ACE2* expression, which promotes viral entry into cells [26]. It also activates proinflammatory responses against viral infection, which can contribute to the cytokine storm [26]. Conversely, histone deacetylase inhibitors have been reported to downregulate *ACE2* and the production of infectious SARS-CoV-2 [26,30], suggesting a potential therapy for COVID-19 (Figure 1). The cytokine storm, which causes many of the adverse health outcomes in COVID-19, could also potentially be regulated through epigenetic modulation [31].

On a broader level, 332 human proteins, some of which are epigenetic regulators, are estimated to strongly interact with SARS-CoV-2 proteins. Any alteration in these proteins results in a deviance from typical cell function and can exacerbate disease conditions [26]. Several epigenetic proteins linked to SARS-CoV-2 have kinase activity and could be targeted using kinase inhibitors. Due to their prevalence in many processes involved in COVID-19 pathogenesis, epigenetic pathways can be potential targets for COVID-19 therapeutics.

## 5. Epigenetic Therapies May Help to Mitigate COVID-19 Severity

Modulation of the epigenetic landscape largely determines differential gene expression in several diseases. Several key genes involved in COVID-19 are impacted by epigenetic pathways [32]. Hence, further research into these pathways would improve our understanding of the disease and epigenetic therapies could be used to mitigate COVID-19. For COVID-19 therapies, the major target genes would be *ACE2*, *TMPRSS2* and Furin to prevent the entry of the virus and cytokines. As mentioned earlier, *ACE2* is the receptor for SARS-CoV-2. *TMPRSS2* and Furin also trigger SARS-CoV-2 infection by cleaving *ACE2*, which promotes viral uptake and allows cell entry [32].

*ACE2* gene expression is downregulated by DNA methylation and histone modification, offering a mechanism for therapy [32]. For example, histone methyltransferase EZH2-mediated H3K27me3 modifications of the *ACE2* promoter could be a target for COVID-19 therapies [33]. In vitro data suggest that vitamin D and quercetin could inhibit *ACE2* and Furin, thereby mitigating COVID-19’s severity [34,35]. However, the prevalence of *ACE2*’s function in physiology, especially in the cardiovascular and renal systems, renders ACE2 inhibitors risky in a clinical setting [36,37]. Hence, further studies on compounds like curcumin and 8-hydroquinone, which may activate DNMTs to silence *ACE2* within viable clinical doses [38,39,40], may be beneficial. Currently, several epigenetic-based clinical trials against COVID-19 are being pursued globally; some of them are listed in Table 1.

## 6. Vitamin D Has a Plausible Protective Effect against COVID-19

The vitamin D endocrine system regulates 3% of the human genome, and it is heavily involved in both innate and adaptive immunity [34]. Active vitamin D is crucial for immune regulation, while its deficiency has been associated with chronic lung diseases [41]. In the airway epithelium, vitamin D controls vitamin D receptor (VDR)-induced gene expression and eliminates pathogens via CD14, antimicrobial peptide mechanisms and the promotion of autophagy [34]. An analysis of 20 patients hospitalized with COVID-19 indicated that 75% had a vitamin D deficiency [42]. Additionally, a study of 43 individuals reported that a combination of vitamin D, magnesium and vitamin B12 was associated with a significant reduction in the proportion of patients who deteriorated [43]. Although the immune functions of vitamin D are known, well-designed trials are necessary to establish a plausible protective role of vitamin D in COVID-19.

## 7. Overview of Metabolic Abnormalities Associated with COVID-19

In addition to epigenetic disruptions, COVID-19 results in metabolic aberrations that can alter the energy metabolism and cause changes in appetite, with the body burning more calories to support the elevated immune response. COVID-19 has been associated with increased insulin resistance, which can negatively impact glucose metabolism [44]. This can lead to changes in blood sugar levels and an increased risk of type 2 diabetes. The immune response to COVID-19 can cause inflammation, which can affect various metabolic processes. Chronic inflammation has also been linked to various health conditions, such as obesity, metabolic syndrome and cardiovascular disease. One of the indirect effects of COVID-19 on metabolism is weight gain.

With many people leading sedentary lifestyles and work routines, especially during the 2+ years of the COVID-19 pandemic, there has been a marked increase in excessive calorie intake and a lack of sufficient physical activity to burn these calories. This dangerous combination has led to weight gain and associated illnesses worldwide. Metabolic and vascular problems were present in up to 50% of COVID-19 fatalities [45]. Furthermore, COVID-19 and the metabolic and endocrine systems have several direct connections. As a result, individuals with metabolic dysfunction (such as obesity, hypertension, non-alcoholic fatty liver disease and diabetes) are not only more likely to develop severe COVID-19, but a SARS-CoV-2 infection may also bring about new cases of diabetes or worsen pre-existing metabolic diseases. COVID-19, in conjunction with type 2 diabetes and obesity, which are both characterized by severe insulin resistance [46], has numerous consequences.

Almost 4 years have passed since the initial outbreak of SARS-CoV-2. Research during this period has revealed that people with metabolic diseases are not only more susceptible to severe COVID-19, but also have an increased risk of post-acute sequelae of COVID-19 and vaccine breakthroughs [47,48,49]. To address these concerns, high-throughput omics-based research on large cohort studies using samples from COVID-19 patients has shed significant light on its etiology, prognosis and outcomes. This has established a new specialization called COVIDomics, which encompasses omics-level research on COVID-19 diagnosis, prevention and biomarkers; the identification of therapeutic targets; and all other aspects associated with SARS-CoV-2 infection [50]. COVIDomics has been explored in a review by Costanzo et al., with a very robust and comprehensive analysis of metabolomics, lipidomics and proteomics studies on plasma, serum and infected cells from COVID-19 patients and a multiomics integrational analysis [50].

## 8. Abnormal Metabolism and Diabetes Are Often Manifested in COVID-19

Several studies present associations between COVID-19 severity, increased mortality, diabetes mellitus and the individual degree of hyperglycemia [51,52,53,54,55,56]. The development of temporary insulin resistance in adults with type 1 diabetes mellitus (T1DM) and type 2 diabetes mellitus (T2DM) has been linked to acute respiratory viral infections like COVID-19, and hyperglycemia has also been linked with severe COVID-19 [56]. Based on a commonly accepted explanation, these individuals are predisposed to the excessive release of cytokines, or a “cytokine storm”, since they experience a state of chronic metabolic inflammation. These elevated levels of inflammatory cytokines might in turn trigger multi-organ failure [56]. The main entry receptor for SARS-CoV-2 is angiotensin-converting enzyme 2 (*ACE2*). The ability of the pancreas to produce insulin in response to hyperglycemia may be impaired when SARS-CoV-2 binds to pancreatic *ACE2* receptors, causing damage to the islets [56].

Many other pathophysiological processes have also been suggested, such as elevated levels of tissue-associated enzymes, the altered expression of ACE2 receptors, immune regulatory dysregulation, pulmonary and endothelium dysfunction, systemic inflammation and hypercoagulability and higher concentrations of anti-inflammatory biomarkers including IL-6, D-dimers and C-reactive protein. All these pathophysiological issues may contribute to an increased response to the cytokine storm that causes inflammation in patients with T1DM or T2DM, which may lead to a more severe COVID-19 course [56].

Additionally, a 2020 analysis of eight cohort studies indicated that COVID-19 patients with excess adiposity had a higher risk of death and serious illness [57]. Obesity and low-grade systemic inflammation are common in individuals with cardiometabolic disorders, and this may be a possible pathway connecting severe COVID-19 with insulin resistance, hypertension, cardiovascular disease and T2DM. The chronic care of patients with T2DM can result in fewer microvascular and macrovascular problems when risk variables such blood pressure, dyslipidemia and glucose levels are managed [58]. There is proof that multifactorial risk factor therapies have a lasting positive impact on mortality, cardiovascular and renal outcomes [58].

The major cause of death from COVID-19 is acute respiratory distress syndrome (ARDS), which develops as a result of an accelerated inflammatory response that releases proinflammatory cytokines including interleukin (IL) and tumor necrosis factor alpha [59]. The family of proteins known as Toll-like receptors (TLRs) serves as sensors and aids the immune system in distinguishing between its own cells and invaders. In the host cell membrane, SARS-CoV-1 and, most likely, SARS-CoV-2 interact with TLR to promote the expression of the main response gene for myeloid 88 (MyD88) differentiation [60]. This then causes nuclear factor kappa beta to become active, ultimately triggering an inflammatory cascade that worsens lung injury [61].

## 9. Hyperglycemia Is Associated with COVID-19 Severity

Chronic hyperglycemia is a condition that impairs both antibody-mediated immunity and innate immunity. Chronic low-grade inflammation may contribute to diabetes by inducing insulin resistance, disrupting glucose control and elevating inflammatory markers [62,63,64]. An increase in IL-6 and C-reactive protein (RCP) levels was observed in diabetic individuals who had the SARS-CoV-2 virus. This proinflammatory state of diabetes led to the release of cytokines and a systemic inflammatory response that accompanied acute respiratory distress syndrome (ARDS) in COVID-19 patients [65,66].

Many of the mechanisms that connect diabetes and hypertension interact in bidirectional ways. First, this complex network is affected by several typical biological factors, including renin–angiotensin–aldosterone system (RAAS) homeostasis, elevated oxidative stress, systemic proinflammatory conditions and enhanced sympathetic nervous system (SNS) activation [67]. The activity of insulin-mediated vasodilator and vasoconstrictor molecules is unbalanced as a result of decreased insulin sensitivity. This results in the vascular system’s remodeling, stiffening and fibrosis, which are predominantly controlled by MAPK-dependent signaling pathways. In fact, insulin increases the production of several vasoconstrictor mediators such as vascular cell adhesion molecule 1, PAI-1 and endothelin-1 [68,69,70].

Additionally, diabetes and the hypertension risk and severity are influenced by obesity and adipose tissue hormone release [71]. Similar to hypertension, excess body fat may change how the pulmonary viral pathogenesis, milieu and immune cell trafficking interact [67,72]. The relationships between many organs and metabolic processes are complicated, but the three primary ones that appear to be at play are inflammation, ACE-2 receptor modulation and hyperglycemia and immune system dysregulation.

In patients with chronic SARS-CoV-2 infection, hyperglycemia has been linked to both illness severity and mortality [71]. These results are remarkably in line with research on patients with highly virulent avian influenza, SARS and MERS, where uncontrolled hyperglycemia was linked to worse outcomes. Numerous biochemical pathways, such as a changed immunological response, an inflammatory echo and the modulation of the virus’s receptor expression utilized for cell entrance, have been hypothesized as links between hyperglycemia and SARS-CoV-2 infection. In fact, elevated blood sugar levels may promote viral entrance and replication in vivo, potentially by changing the *ACE2* receptor [72,73,74]. Increased glucose levels may also inhibit the immune system’s ability to fight viruses, making viral infections more severe.

Reduced neutrophil degranulation, phagocytic activity and chemotaxis inhibit the lymphocyte proliferative response and impair the complement activation of the innate and adaptive immune responses, all of which are affected by hyperglycemia [75,76]. It is believed that diabetes-related hyperglycemia impairs the immune system’s ability to prevent the spread of infection in diabetic people [76]. According to a study, hyperglycemia significantly lowers the macrophagic activity of both macrophages and neutrophils, leaving patients more vulnerable to infection [77]. High glucose levels are associated with reduced vascular dilatation and increased permeability during the early stages of inflammatory reactions, potentially as a result of protein kinase C activation [78].

**Figure 2:**
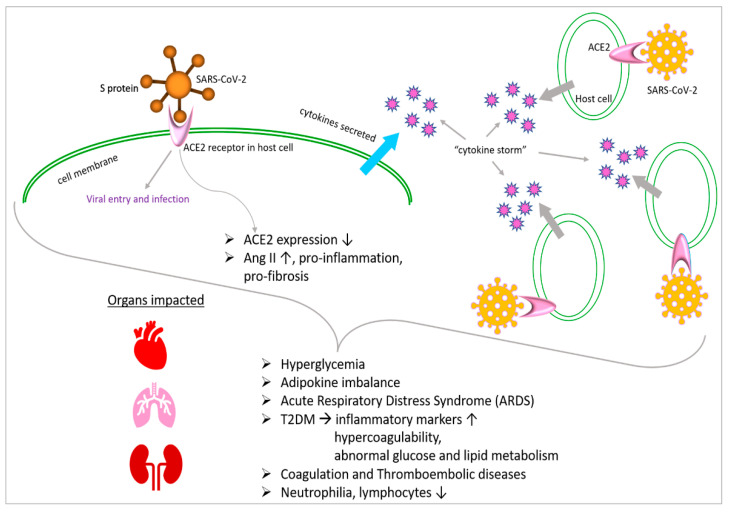
Metabolism-associated impacts of SARS-CoV-2 infection. SARS-CoV-2 S protein interacts with ACE2 receptor on host cell membrane, leading to viral entry and infection. Significant downstream events are reduced expression of *ACE2* and cytokine storm. Metabolism-associated phenotypes and adversely impacted organs are listed. ↓—upregulation, ↑—downregulation.

Additionally, hyperglycemia can directly glycolyze proteins and affect the tertiary structure of complements. These modifications reduce phagocytosis and impair the immunoglobulin-mediated opsonization of bacteria, as well as complement fixation to bacteria [78]. As a result, the evidence points to a dysregulated immune response as the likely cause of the higher disease severity seen in people with SARS-CoV-2 infection and associated hyperglycemia. This leads to a more severe and extended pathology. A substantial link between hyperglycemia and a worsened result from SARS-CoV-2 has also been demonstrated. COVID-19 individuals with T2DM are reported to be more prone to having increased inflammatory markers, hypercoagulability and abnormalities of glucose and lipid metabolism (Figure 2). They are also more likely to have hypoproteinemia [79].

## 10. COVID-19 Affects Adipokines with Impacts on Glucose Metabolism

In order to effectively regulate the clinical outcomes in infected patients with coexisting obesity, hyperglycemia and diabetes, a glycemic profile must be established in SARS-CoV-2 patients. As previously mentioned, obesity may be another relevant factor linked to a poor prognosis in COVID-19 patients. Adipose tissue and the immune system’s intricate communication may be significant to SARS-CoV-2 infection. Although immune cells are present throughout the adipose tissue in the normal state, adipocytes and immune cells are in a state of equilibrium, which in turn results in the synthesis of adipokines [80].

A large number of immune cells invade the adipose tissue in pathophysiological circumstances like obesity, which causes an imbalance in the synthesis of adipokines, including leptin and adiponectin [81,82]. Additionally, via raising insulin sensitivity and glucose uptake and thus raising GLUT-4 translocation, these adipokines are implicated in the glucose balance, energy homeostasis and insulin sensitivity [81,83]. Understanding the processes behind the connections between comorbid diseases, immunological dysregulation and hormones generated by adipocytes may help us to better understand how the SARS-CoV-2 pathogenesis emerges.

## 11. COVID-19 Affects Metabolism through Interactions with Angiotensin

A spike (S) protein that protrudes from the viral envelope is responsible for the attachment and adherence of coronaviruses to host or human cells [53,84]. It has been confirmed that the S1 (subunit of the SARS-CoV-2 spike protein) region of SARS-CoV and SARS-CoV-2 binds to angiotensin-converting enzyme 2 (Figure 1). This interaction occurs through the receptor-binding domain (RBD) of S1. The mono-carboxypeptidase *ACE2* was first discovered as an ACE receptor homolog in 2000 [85,86], and, since then, its molecular structure has been completely characterized [87]. A wide range of biological systems, including bladder urothelial cells, kidney proximal tubule cells, cholangiocytes, enterocytes, esophageal epithelial cells, myocardial cells and type II lung alveolar cells, express *ACE2* [88]. By cleaving a single amino acid in the human lung, *ACE2* produces angiotensin (I–VII) from angiotensin II [89]. Ang (angiotensin)-(I–VII), through Mas receptor (Mas1) activation, is expressed on endothelial cells and results in vasodilatory, anti-inflammatory and antifibrotic effects [90]. It is interesting to note that the interaction between SARS-CoV-2 and ACE2 causes *ACE2* expression to be downregulated, which causes the aggregation of AngII (angiotensin II) with proinflammatory and profibrotic effects [91,92].

In a limited cohort study, plasma samples infected with SARS-CoV-2 were shown to have significantly higher levels of Ang II [93]. Apoptotic stimuli could be activated by many signaling pathways. First, it has been discovered that elevated oxidative stress, which is caused by a hyperactive AngII/AT1R/NAPDHox axis, is linked to cardiovascular diseases, including hypertension and atherosclerosis [94,95]. Hence, the production of reactive oxygen species (ROS) downstream triggers the release of CytC from damaged mitochondria [96], the p38MAPK/JNK cascade or the activation of caspase 3 [97], all of which are known to induce apoptosis. Additionally, proapoptotic signals have also been linked to nuclear factor kappa B (NF-kb) activation and the production of cytokines such as interleukin-6, IL-1 and tumor necrosis factor alpha (TNFα) [98]. When cyclo-oxygenase 2 (*COX2*) is elevated due to high levels of AngII and proinflammatory cytokines, ROS and inflammatory prostaglandin E2 are produced as a result [99].

## 12. Metabolism-Related Therapeutics Could Be Promising against COVID-19

It is interesting to note that polymorphisms for *ACE2* were independently linked to a higher risk of developing hypertension and cardiovascular problems in people with diabetes [100,101]. In the late stage of SARS-CoV-2 infection, the overactivation of these pathways can lead to a condition of hyperinflammation. Agents that operate on the renin–angiotensin system (RAS) noticeably alter *ACE2* expression. The foundation of many therapies for cardiovascular and renal illnesses is the use of RAS inhibitors. To stop diabetic nephropathy and cardiovascular remodeling, these medicines are frequently prescribed to diabetic patients. Different responses to the administration of agents that interfere with this regulatory axis have been observed in experimental and clinical models. In particular, angiotensin II receptor blockers (ARBs) and mineralocorticoid receptor blockers appear to increase the levels of *ACE2* expression [102,103], whereas the administration of ACE inhibitors, which increased cardiac *ACE2* mRNA levels, did not result in higher ACE2 activity [103]. In a different study, olmesartan-treated hypertension patients had higher urine *ACE2* levels [104].

However, several international societies and associations have advised against stopping angiotensin-converting enzyme inhibitors (ACE-Is) or sartans in patients receiving long-term therapy, based on the previously mentioned mechanisms connecting *ACE2* expression with local antiproliferative, antifibrotic and anti-inflammatory properties [105]. Other diabetes medications may inhibit the RAS’s ability to function normally. An important family of insulin sensitizers used in the treatment of T2DM is thiazolidinediones. The modification of peroxisome proliferator-activated receptors mediates the molecular processes of the biological reactions to thiazolidinediones in diabetic patients (PPARs). Thiazolidinediones, like ACEIs and ARBs, increase *ACE2* expression [106,107], which could expose alveolar cells to SARS-CoV-2 infection. PPARs are inflammatory mediators with possible immunoregulatory properties. The inflammatory cytokines IL-6 and INF, which are heavily connected in SARS-CoV-2, are reduced as a result of their activation [108].

**Figure 3:**
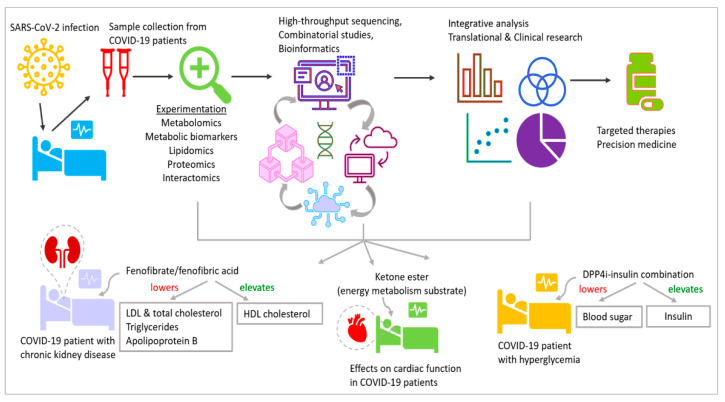
Importance of COVID-19 metabolomics research in therapeutic development. Metabolomic and multiomic studies along with integrative analysis of samples collected from COVID-19 patients significantly contribute to translational and clinical research for therapeutic development. LDL—low-density lipoprotein, HDL—high-density lipoprotein, DPP4i—dipeptidyl peptidase-4 inhibitor; ketone ester—d-β-hydroxybutyrate-(*R*)-1,3 butanediol monoester.

Insulin-mimetic medications like rosiglitazone and pioglitazone, which are used to treat T2DM, have a significant overall ability to reduce influenza virus infection [108]. The cardiovascular and renal advantages of sodium-glucose cotransporter 2 inhibitor (SGLT2) therapy for those with T2DM are now also established, being applied those without T2DM, according to studies [58]. Given the pathophysiology of COVID-19 and the benefits of SGLT2 inhibitors and glucagon-like peptide-1 receptor agonists (GLP1RA) that have been documented, these treatments may be preferable to other therapeutic options for patients with T2DM and long COVID, and maybe even for those without diabetes mellitus. Therapeutic interventions towards metabolism are implemented in several clinical trials against COVID-19, e.g., NCT04517396, NCT04542213 and NCT04573764 (Figure 3 and Table 1).

## 13. Inflammatory Immune Response in Diabetic COVID-19 Patients Is Deleterious

It has been established that infectious diseases significantly increase mortality in diabetes patients by linking acute viral respiratory infection to the fast development of transitory insulin resistance in both overweight individuals and healthy euglycemic normal-weight individuals [109,110]. Older diabetes patients have higher death rates, according to a retrospective review [111]. Due to a combination of dysregulated innate immunity and inflammatory responses, diabetes is closely linked to an elevated risk and worse outcomes for bacterial and viral infections [112,113].

The gastrointestinal tract and impaired epithelial barrier function in the lungs of diabetic people also make those with coronavirus infection susceptible to subsequent bacterial infections [109]. The death of patients affected by SARS-CoV-2 is a result of chronic inflammation caused by the synthesis of related cytokines during viral infection. These conditions include coagulation activation, neutrophilia and kidney damage [65,66,114]. Several studies have shown that, in diabetic patients with SARS-CoV-2, the absolute count of lymphocytes in the peripheral blood is significantly lower, while the absolute count of neutrophils is remarkably higher [115].

Additionally, compared to non-diabetic patients with SARS-CoV-2, diabetic individuals had increased serum levels of inflammatory-related biomarkers [80]. These people are distinguished particularly by increased blood levels of IL-6, TNF-α, C-reactive protein (CRP) and serum ferritin. Among them, IL-6 has a longer expression duration than other cytokines (IL-1 and TNF-α) and is a predictor of the severity of illness and prognosis [116]. Additionally, it is also observed that there is an increase in serum ferritin in diabetes patients, confirming the activation of the monocyte–macrophage system, an essential component of the inflammatory storm [65,66,116].

## 14. Immunometabolic Phenotyping Reveals T Cell and Myeloid Cell Populations Unique to Severe COVID-19

The factors that determine why some patients quickly recover from SARS-CoV-2 infection while others experience severe disease, potentially leading to death, remain unclear. Previous research has identified associations between COVID-19 and generalized changes in immune cell subsets [117]. However, these studies do not consider host deficiencies specific to SARS-CoV-2 as opposed to other viral infections [118]. Furthermore, investigating the reciprocal interaction between metabolic reprogramming and immune function in the context of COVID-19 pathogenesis could provide novel insights. To examine metabolic programs at the single-cell level, a flow-cytometry-based proteomic and epigenetic approach was utilized. Samples of peripheral blood mononuclear cells (PBMCs) from individuals with acute COVID-19 and recovered individuals were compared to those of healthy controls, individuals hospitalized with influenza, individuals with acute hepatitis C and individuals with chronic hepatitis C.

In an unbiased analysis of T cells employing combined immune and metabolic markers, a population of T cells unique to patients with acute COVID-19 was identified. This population exhibited the upregulation of voltage-dependent anion channel 1 (VDAC1)—a mitochondrial membrane protein involved in metabolite transport and mitochondrial cell death signaling [119]—and the upregulation of histone H3 lysine 27 trimethylation (H3K27me3), which is an epigenetic modification for transcription repression [118]. Interestingly, this subset of cells also expresses low levels of the glucose transporter 1 and hexokinase II (HKII) proteins, which are typically upregulated alongside VDAC1 and H3K27me3 [6], and higher levels of translocase of outer mitochondrial membrane 20 (Tomm20) and killer cell lectin-like receptor subfamily G member 1 (KLRG1, associated with T cell senescence) [120,121]. To determine whether elevated VDAC1 and Tomm20 indicate altered mitochondrial function, electron microscopy was performed on PBMCs from acute COVID-19 patients and healthy controls. In severe COVID-19 patients, the mitochondria were irregularly shaped, and cytochrome c was found in the cytosol (subsequently inducing apoptosis), suggesting that high VDAC1 expression makes these T cells more susceptible to death. However, this apoptosis was inhibited in vitro by targeting VDAC1 oligomerization.

The study also found that the prevalence of the unique T cells increased with age (an established risk factor for severe COVID-19). These results suggest that targeting mitochondrial dysfunction could be a possible course for treatment. The study also used an immunometabolic assay to examine myeloid cells in PBMC samples, which showed increased inflammation and activation in COVID-19 patients in previous studies [122]. Myeloid-derived suppressor cells with metabolic phenotypes unique to severe COVID-19 were identified. The frequency of these cells was positively associated with the COVID-19 severity, suggesting that they may indicate dysregulated inflammation. These results provide more cell-specific insights into the factors associated with COVID-19’s severity and distinguish subsets of immune cells that could help to predict disease severity and be used as metabolic targets for future treatment.

## 15. Hyperglycemia Associated with COVID-19 Impacts Blood Coagulation

Hyperglycemia is a significant risk factor for a defective coagulation balance and platelet aggregation, which may contribute to the worsened thromboembolic diseases seen in deceased COVID-19 individuals. In diabetic patients, a number of mechanisms have been investigated that connect inflammation with coagulative homeostasis [114]. First, inflammation activates plasmin, which increases D-dimer. Second, severe inflammation and hypoxia activate monocyte–macrophages, and thrombin activation results in the secretion of a large number of tissue factors and the activation of the exogenous coagulation pathway, which results in a general hypercoagulable state or even disseminated intravascular coagulation [65,66,114].

Furthermore, increased D-dimer levels are frequently observed, and they show a steady increase when the disease begins to worsen [123]. Thus, the longitudinal analysis of lymphocyte count dynamics and inflammatory markers like IL-6, CRP and ferritin over the course of the disease may assist in the detection of people with a poor prognosis and the initiation of prompt treatment to improve outcomes.

## 16. Discussion

Several studies have demonstrated the impact of COVID-19 on epigenetic and metabolic pathways. Due to the direct impacts of epigenetics and metabolism on development, these studies can help us to better understand the potential impact of COVID-19 on development. For example, major epigenetic signatures like DNA methylation and histone modifications are impacted by COVID-19, which influence gene expression profiles and accelerate aging. This can contribute to abnormal protein turnover and multi-organ failure, ultimately leading to death. The metabolic impacts are also significant; the combined epigenetic and metabolic alterations impact inflammation and the upregulated immune response seen in COVID-19. In COVID-19 patients hospitalized with symptoms, the unregulated production of IL-6 has been shown to correlate with the illness severity and progression [124] and predict respiratory failure [125]. This is noticeable in diabetic patients, where low-grade inflammation (characteristic of this pathology) can facilitate the cytokine storm caused by SARS-CoV-2 infection. Within the storm, the released IL-6, cytokines, TNF-α and IL-1B can have a major impact on glucose metabolism and insulin signaling and induce cardiovascular complications [126]. The secretion of IL-6 can also raise CRP [127]. The activation of complements and tissue factors that starts coagulation can be caused by CRP aggregates [128].

Current studies also highlight the increased likelihood of diabetics to develop coagulopathies such as disseminated intravascular coagulation (DIC) [129], a condition that might be worsened by SARS-CoV-2 infection and have serious consequences [124]. The rise in IL-6 that accompanies COVID-19 may prevent autophosphorylation and activate the phosphoinositide 3 kinase (PI3K) [130] and protein kinase B (AKT) pathways, thereby worsening insulin resistance in diabetics [131,132]. Increased TNF-α levels have also been demonstrated to cause insulin resistance by affecting insulin signaling and glucose absorption in vivo, similarly to IL-6 [133]. TNF-α can cause NF-kb to translocate into the nucleus and encourage the transcription and release of more cytokines, resulting in a vicious circle. Additionally, mortality and B cell malfunction may occur due to the excessive production of IL-1B, which can produce excessive amounts of nitric oxide [134].

Leptin, a proinflammatory cytokine that is chronically elevated in obese patients, has been identified as a cofactor in the severity and progression of AH1N1 influenza that results in acute lung injury [135]. It is also believed to be a major factor in the development of hypertension, cardiovascular diseases and insulin resistance [136]. Further, decreased immune responses in obese individuals might result in vaccine failure, delaying the recovery from a viral infection [137]. It is not surprising that diabetes is a risk factor for SARS-CoV-2 infection given the effect that proinflammatory cytokines can have on insulin signaling and glucose metabolism. Additionally, it is likely that healthy individuals who have been severely impacted by COVID-19 are susceptible to the development of diabetes due to an extended and acute increase in proinflammatory cytokines. Overall, studies show that COVID-19 affects different aspects of metabolism with adverse outcomes [138,139,140,141].

Collectively, the available information establishes a strong foundation for further research in epigenetics and metabolism associated with viral infections, involving large cohorts of patients and utilizing cutting-edge multiomcs coupled with next-generation sequencing and advanced bioinformatics analysis [7,9,50,142,143,144]. These studies will contribute to targeted therapeutic development and precision medicine (Figure 3). As with all other promising technologies, the current studies need to overcome challenges in terms of effectiveness, which can be achieved by further research in the future. The results will reveal further insights into the molecular mechanisms at play and answer the questions in developmental biology that are currently unanswered.

## 17. Future Perspectives: Potential Aims and Experimental Methods

The aim of this review is to present the latest findings on the metabolic and epigenetic disruptions impacting health and development when the immune system is severely impacted in COVID-19. The methods and results from the studies discussed could help us to prepare better for diseases with similar manifestations in terms of prevention and cures. The quest for a cure for COVID-19, and other infectious diseases manifesting a cytokine storm and leading to organ damage and death, forces us to focus on the correlation between inflammation and resistance to cisplatin, which is a therapeutic but also induces proinflammatory signaling and toxicity [145]. This focus stems from the failure of the response and severe inflammation associated with the drug. The most common cisplatin-induced toxicities (CITs) are nephrotoxicity (CIN), peripheral neuropathy (CIPN) and ototoxicity (CIO), which are associated with persisting markers of inflammation. In cancer, one of the applications of cisplatin is to activate and release extracellular vesicles from tumor cells, which becomes more complicated because cancer cells often become resistant to cisplatin and, hence, a combination of drugs needs to be administered [146]. In COVID-19, similar complications can be anticipated because a cytokine storm is observed and studies in cancer have shown that the signaling pathway of an inflammatory cytokine called interleukin 1β (IL-1β) contributes to the cisplatin resistance of cancer cells [147].

To address these challenges, studies have focused on combination treatments. One study has shown that cisplatin susceptibility in tumors can be achieved by the inhibition of transient receptor potential vanilloid 1 (TRPV1), which is a nonselective cationic channel regulating Ca^2+^ influx [148]. Interestingly, various tissues like the brain, kidney, bronchial epithelial cells and others show the wide expression of TRPV1, and these organs are damaged by the severe cytokine storm of COVID-19. Further, TRPV1 is associated with pain sensations, autophagy, inflammation and apoptosis, which are processes that show abnormalities in COVID-19. In support of combinatorial therapies for cisplatin resistance, a study has focused on MAST1, which is a critical platinum resistance factor that reprograms the mitogen-activated protein kinase pathway for signal transduction. The study showed that the inhibition of MAST1 by lestaurtinib abrogated tumor growth in circumstances where the co-administration of dexamethasone and cisplatin reinstated cisplatin-resistant tumor growth [149]. This observation provides beneficial information when designing methods for therapeutic interventions against COVID-19 and other infectious diseases with similar inflammatory manifestations.

In further support of combinatorial therapies targeting various signal transduction pathways, a study on gastric cancer showed that cisplatin resistance can be overcome by inhibiting the axis involving interleukin 6 (IL-6) and signal transducer and activator of transcription 3 (STAT3), in combination with cisplatin [150]. Cisplatin resistance is defined as a driver of poor prognosis; hence, this study is yet another important example that emphasizes the need for co-intervention with multiple therapeutics instead of a single agent. In a similar direction, an immunosuppressive medication called tocilizumab has been used in COVID-19 research. While one study showed that hospitalized COVID-19 patients with hypoxia and systemic inflammation experienced improvements in survival and other clinical outcomes upon the administration of tocilizumab [151], another study found no significant improvements [152].

A recent study showed that moderate to severe COVID-19 patients benefitted more from the administration of tocilizumab and another immunosuppressive medication called infliximab, in addition to the standard treatment [153]. However, one therapeutic agent may not be sufficient to improve multifarious outcomes in various stages of a disease. For example, iron metabolism, the rate of anemia and sepsis occurrence in critically ill COVID-19 patients did not show differences with or without tocilizumab treatment [154]. Indeed, tocilizumab significantly decreases respiratory support requirements and inflammatory markers in COVID-19 patients, but it only reduces mortality if administered at the early inflammatory stages and in high doses in severely ill patients [155]. Collectively, these studies provide valuable information on experimental methods, potential therapies and future perspectives towards developing efficient preventative and therapeutic procedures against COVID-19 and similar diseases.

## Figures and Tables

**Table 1:** Clinical trials investigating various interventions for COVID-19: a summary.

NCT *	Intervention/Aim	Clinical Trial Description
NCT04411563	Predicting prognosis markers	Quantification of circulating epigenetic factors, e.g., microRNAs, profiling of DNA methylation
NCT04859894	Correlating symptoms and physiology of COVID-19 patients with epigenetics	Studying epigenetic alterations and DNA methylation patterns
NCT04939155	Assess effects of SARS-CoV-2 infection and vaccination	Analyze epigenomes and DNA methylation pre-/post-infection and vaccination
NCT04517396	Fenofibrate/fenofibric acid. Lowers elevated LDL and total cholesterol, triglycerides, apolipoprotein B. Increases HDL cholesterol	Fenofibrate in chronic kidney disease to improve clinical outcomes in COVID-19
NCT04542213	Dipeptidyl peptidase 4 inhibitor (DPP4i). Elevates insulin, lowers blood sugar	DPP4i–insulin combination for metabolic control and prognosis in hospitalized patients with SARS-CoV-2 and hyperglycemia
NCT04573764	d-β-hydroxybutyrate-(*R*)-1,3 butanediol monoester. Ketone body, maintains ATP yield at starvation, more efficient substrate for energy metabolism than glucose	Acute effects of oral ketone ester on cardiac function in COVID-19 patients

* National Clinical Trial.

## Data Availability

Not applicable.
